# Supplementary figures and images for: Effects of hydraulic retention time and influent nitrate concentration on solid-phase denitrification system using wheat husk as carbon source
Source: PeerJ. 2023 Jul 24;11:e15756. doi: 10.7717/peerj.15756 (PMC10373648; doi:10.7717/peerj.15756)

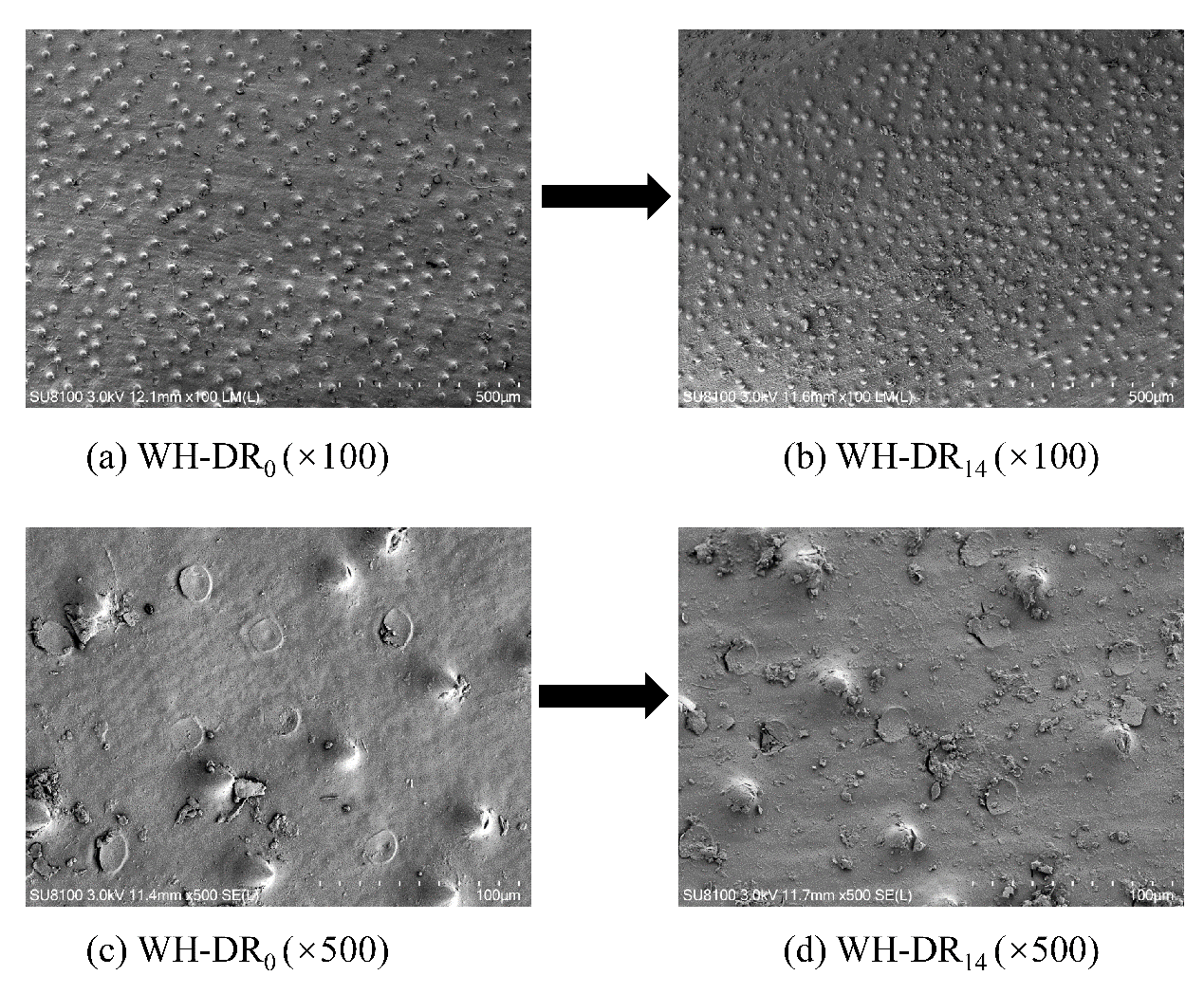

Supplement: Supplemental Information 1 [file peerj-11-15756-s001.png]
