# Supplementary material for: Effects of hydraulic retention time and influent nitrate concentration on solid-phase denitrification system using wheat husk as carbon source
Source: PeerJ. 2023 Jul 24;11:e15756. doi: 10.7717/peerj.15756 (PMC10373648; doi:10.7717/peerj.15756)
Supplement: Supplemental Information 2 [file peerj-11-15756-s002.docx]

Table S1 Composition of artificial synthetic culture wastewater

| Component | Concentration (mg/L) |
| --- | --- |
| KNO_3_ | 360 |
| NH_4_Cl | 21.2 |
| NaNO_2_ | 12.3 |
| K_2_HPO_4_ | 78 |
| KH_2_PO_4_ | 44 |
| MgSO_4_ ∙7H_2_O | 44 |
| KCl | 37 |
| Trace elements 0.2% (V/V) |  |
| EDTA | 640 |
| FeSO_4_ ∙7H_2_O | 550 |
| ZnSO_4_ ∙7H_2_O | 230 |
| MnSO_4_ ∙H_2_O | 340 |
| CuSO_4_ ∙5H_2_O | 75 |
| Co(NO_3_ )_2_ ∙6H_2_O | 47 |
| (NH_4_)_6_ Mo_7_O_24_ ∙4H_2_O | 25 |
