# Supplementary material for: Effects of hydraulic retention time and influent nitrate concentration on solid-phase denitrification system using wheat husk as carbon source
Source: PeerJ. 2023 Jul 24;11:e15756. doi: 10.7717/peerj.15756 (PMC10373648; doi:10.7717/peerj.15756)
Supplement: Supplemental Information 3 [file peerj-11-15756-s003.docx]

Table S2 Analysis results of main components of WH before and after experiment

| Sample | lignin | cellulose | hemicellulose | C | N | P |
| --- | --- | --- | --- | --- | --- | --- |
| WH-DR_0_ | 15.99% | 41.96% | 25.39% | 49.10% | 0.89% | 0.43% |
| WH-DR_14_ | 15.62% | 41.30% | 24.70% | 48.46% | 0.87% | 0.41% |
